# Role of the multiple efflux pump protein TolC on growth, morphology, and biofilm formation under nitric oxide stress in *Cronobacter malonaticus*

**DOI:** 10.3168/jdsc.2020-0040

**Published:** 2021-03-19

**Authors:** Dengyu Liu, Yaping Wang, Xin Wang, Dexin Ou, Na Ling, Jumei Zhang, Qingping Wu, Yingwang Ye

**Affiliations:** 1 School of Food and Biological Engineering, Hefei University of Technology, Hefei, 230009, China; 2 Guangdong Provincial Key Laboratory of Microbial Culture Collection and Application, State Key Laboratory of Applied Microbiology Southern China, Guangdong Institute of Microbiology, Guangdong Academy of Sciences, Guangzhou, 510070, China

## Abstract

Nitric oxide (NO) is a biological signal molecule that can control and prevent the growth of most pathogens. *Cronobacter* species are a group of gram-negative foodborne pathogens that cause severe diseases, including neonatal meningitis, septicemia, and necrotizing enterocolitis, especially among newborns and infants consuming contaminated powdered infant formula. *Cronobacter* species might be tolerant to NO, resulting in severe infections. However, the specific mechanism of tolerance to NO in *Cronobacter* species is unclear. Here, we explore the effects of a key component, the protein TolC, of a multiple efflux pump on the growth, morphological changes, and biofilm formation of *Cronobacter malonaticus* under NO stress. We found that deletion of *tolC* resulted in a decreased growth rate under 100 m*M* sodium nitroprusside (NO donor) and led to more disruptive morphological injury to the bacterial cells. Furthermore, *C. malonaticus* lacking the TolC protein (Δ*tolC* mutant) showed weaker biofilm formation than the wild-type strain under normal or NO stress conditions. We have proved that TolC plays an important role in cell growth and biofilm formation of *C. malonaticus*. Therefore, our results may provide valuable theoretical basis for formulating clinical guidelines for treatment of disease caused by *C. malonaticus* and ensuring food safety.

## Highlights

• Protein TolC contributes to tolerance to nitric oxide stress in *Cronobacter malonaticus*.
• Nitric oxide could inhibit the growth of *Cronobacter malonaticus*.
• Nitric oxide morphologically damaged *Cronobacter malonaticus*.
• Nitric oxide had a negative effect on biofilm formation by *Cronobacter malonaticus*.

## Graphical Abstract

**Figure:**
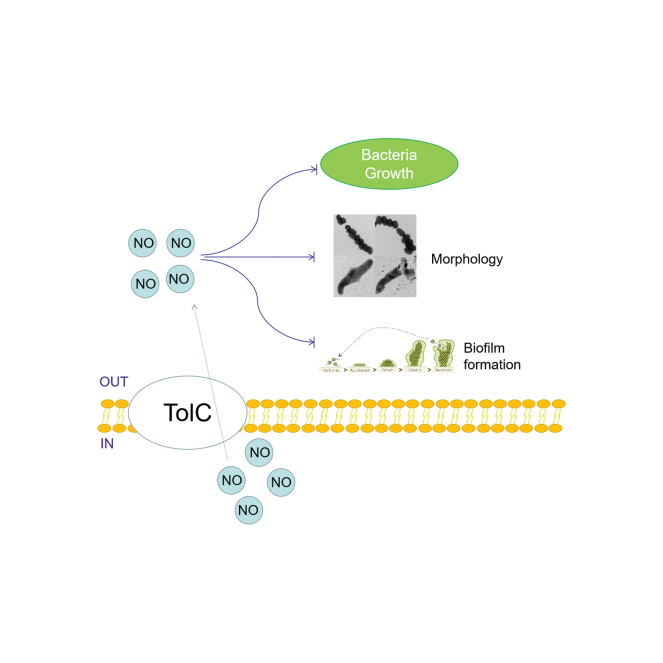
**Summary:***Cronobacter* species are a group of gram-negative foodborne pathogens that cause severe diseases, including neonatal meningitis, septicemia, and necrotizing enterocolitis, especially in newborns and infants consuming contaminated powdered infant formula. In this study, we demonstrated that TolC, a protein component of a multidrug efflux pump in gram-negative bacteria, significantly contributes to tolerance to nitric oxide stress in *Cronobacter malonaticus*, providing a new perspective on prevention and control of infections caused by *Cronobacter* species.

*Cronobacter* species are opportunistic pathogens associated with severe diseases in neonates, including necrotizing enterocolitis, bacteremia, meningitis, and brain abscess or lesions (Bowen and Braden, 2006; Drudy et al., 2006). *Cronobacter* can be divided into 7 species: *C. sakazakii, C. turicensis, C. muytjensii, C. dublinensis, C. universalis, C. condiment*, and *C. malonaticus* (Iversen et al., 2008; Joseph et al., 2012). *Cronobacter malonaticus* can be isolated from clinical samples and different matrices such as infant formula, retail foods, and environmental samples (Fei et al., 2015; Killer et al., 2015; Brandão et al., 2017). Therefore, it is important to understand the molecular mechanism underlying virulence and tolerance of *Cronobacter* spp. to adverse stress to reduce its negative effects.

Nitric oxide (NO) is a gaseous signal molecule with multiple functions in humans, including involvement in the human immune system (Urbano et al., 2018). In the immune system, NO is usually produced by inducible nitric oxide synthase (**iNOS**), and its production is transcriptionally induced by upregulation of iNOS in response to cytokines and microbial products (Bogdan, 2015). During microbial pathogenic infection, high output of NO produced by iNOS can dramatically alter microbial metabolism and physiology (MacMicking et al., 1997; Fang, 2004; Lee et al., 2017).

To counter NO from the immune system, foodborne pathogens use a variety of detoxification systems, including detoxification genes and efflux pumps (Blair et al., 2015). The multidrug efflux pump AcrAB-TolC is present in gram-negative bacteria, and it reduces susceptibility to antimicrobial agents such as colicin E1, hemolysin, and enterotoxins (Mu et al., 2020). In this efflux pump, TolC is an envelope protein, which was named because its loss by mutation conferred tolerance to specific colicins and bacteriophage (Koronakis et al., 2004). TolC plays vital roles in many gram-negative bacteria, such as *Salmonella* (Raspoet et al., 2019), *Escherichia coli* (Thanassi et al., 1997), *Klebsiella pneumoniae* (Iyer et al., 2019), and *Enterobacter aerogenes* (Masi et al., 2007). Although TolC exists in all 7 major species of *Cronobacter*, no research has characterized the functions of TolC in *Cronobacter* spp.

As a major antimicrobial substance, NO has been widely studied in bacteria. To date, little information is available about the roles of TolC in *C. malonaticus*. In this study, we investigated the roles of TolC when *C. malonaticus* is under stress from NO. We determined the growth conditions, morphological changes, and biofilm formation between wild type (**WT**) *C. malonaticus* and a mutant strain of *C. malonaticus* with deletion of the TolC protein (**Δ*tolC*** mutant) under sodium nitroprusside (**Snp**), a nonenzymatic source of NO.

The WT and Δ*tolC* strains of *C. malonaticus* were acquired from the Guangdong Microbiology Culture Center (GDMCC; Guangzhou, China). Both strains were routinely grown in Luria-Bertani (**LB**) broth medium (Huankai) overnight at 37°C, with agitation at 200 rpm for 16 h to prepare the cells for each experiment.

Overnight cultures of *C. malonaticus* WT and Δ*tolC* were diluted 100-fold into 5 mL of LB and 5 mL of LB with 100 m*M* Snp (Guangzhou Chemical Reagent Factory). Then, 200 µL of diluted cultures were added to 96-well plates, and the plates were incubated in a Cytation5 Cell Imaging Multi-Mode Reader (Biotek) with agitation at 200 rpm for 24 h. Absorbance was measured by optical density (**OD**) at 600 nm (OD_600_) every 30 min.

To detect the relative change in cell number, strains were incubated in LB and LB with 100 m*M* Snp, respectively, at 37°C, with shaking at 200 rpm, for 0.5, 1.5, and 2.5 h. The number of cells of the 2 strains at each timepoint under different media was counted using a colony counting method. The relative change in cell number was calculated as the number of cells in the LB broth divided by the number of cells in the cultures with 100 m*M* Snp. Each experiment was done in triplicate.

The strains were incubated in LB and LB with 100 m*M* Snp, and the cells were harvested to detect morphologic changes using transmission electron microscopy (model S-3000N; Hitachi). The treatment procedure was performed according that described by Wang et al. (2013).

For detection of biofilms by crystal violet staining (**CVS**) assay, overnight cultures of the 2 strains were diluted 100-fold into fresh LB and LB with 100 m*M* Snp. Then, 200 µL of diluted culture was added to 96-well plates, and the plates were incubated at 37°C for 24, 48, and 72 h. The plates were then washed 3 times with sterile saline to remove planktonic bacteria, and the adherent bacterial cells were stained with 1% crystal violet for 30 min. After being washed 3 times with sterile saline, the crystal violet was liberated by 33% acetic acid following a 15-min incubation. The OD values of each well were measured at 590 nm. Each experiment was done in triplicate.

For scanning electron microscopy, glass coverslips were placed into 24-well plates containing 1.98 mL of LB or LB with 100 m*M* Snp. Then, 20 µL of overnight culture of each strain was added to wells in a 24-well plate and incubated at 37°C for 24, 48, and 72 h. The glass coverslips from different incubation times were prepared for scanning electron microscopy, as described by Wang et al. (2013).

For confocal laser scanning microscopy (**CLSM**), glass coverslips were prepared in the same manner as for scanning electron microscopy, and bacterial biofilms on glass slips were stained using the LIVE/DEAD BacLight bacterial viability kit according to the manufacturer's instructions (Invitrogen/Thermo Fisher Scientific), and were then observed by CLSM.

*Cronobacter* spp. can tolerate different environmental stresses, such as acid, desiccation, and osmotic and oxidative stress (Yang et al., 2015; Zhang et al., 2018, 2019; Zhou et al., 2020). In this study, we first investigated the tolerance of *C. malonaticus* to NO stress. As shown in Figure 1 (A, B), Snp inhibited the growth of both the WT and Δ*tolC* strains of *C. malonaticus*. Nitric oxide, a key molecule produced by the immune system in mammals, is reported to control the growth of different types of bacteria, including *Pseudomonas aeruginosa* (Hibbard and Reynolds, 2019), *Escherichia coli* (Sivaloganathan and Brynildsen, 2020), and *Staphylococcus aureus* (Urbano et al., 2018). Moreover, NO has been shown to protect microorganisms from harmful reactive oxygen species produced by the immune system or various antibiotics (Gusarov and Nudler, 2005; Gusarov et al., 2009). In the current study, we found no difference between WT and Δ*tolC C. malonaticus* in LB broth (no Snp; Figure 1C), but the WT strain grew better than the Δ*tolC* strain in LB broth with 100 m*M* Snp (Figure 1D). Furthermore, as shown in Figure 1E, the relative change in cell number of WT and Δ*tolC C. malonaticus* treated with 100 m*M* Snp over 0.5, 1.5, and 2.5 h were 58.28 ± 3.95% and 55.15 ± 2.38%, 14.05 ± 1.01% and 8.35 ± 0.31%, and 6.57 ± 0.48% and 0.83 ± 0.10%, respectively, compared with those in LB without Snp. The AcrAB-TolC multidrug efflux pump system is one mechanism leading to bacterial resistance to antibiotics in gram-negative bacteria (Krishnamoorthy et al., 2013). TolC (but not the other part of efflux pump system, AcrB) is essential for colonization of chicks with multidrug-resistant *Salmonella enterica* serotype Typhimurium, which indicates that TolC might be a better target than AcrB for the development of efﬂux pump inhibitors (Baucheron et al., 2005). Overexpressing *tolC* from an inducible plasmid in a low-level resistance mutant of *E. coli* resulted in a higher level of nitroxoline resistance (Puértolas-Balint et al., 2020). When treated with 100 m*M* Snp, both strains in that study showed morphological injuries, including cell lengthening and breaks in the cell membrane. In our study, injuries to cells were more severe in Δ*tolC* than in WT, as shown in Figure 2; cell integrity and cell membranes were heavily damaged in Δ*tolC*, whereas the cell membrane of WT was only slightly damaged. In *Helicobacter pylori*, NO caused a rapid and dose-dependent morphologic conversion of *H. pylori* from the replicating spiral form to the nonreplicating coccoid form (Cole et al., 1999). Our results suggest that TolC is essential for growth of *C. malonaticus* under NO stress.

**Figure 1:**
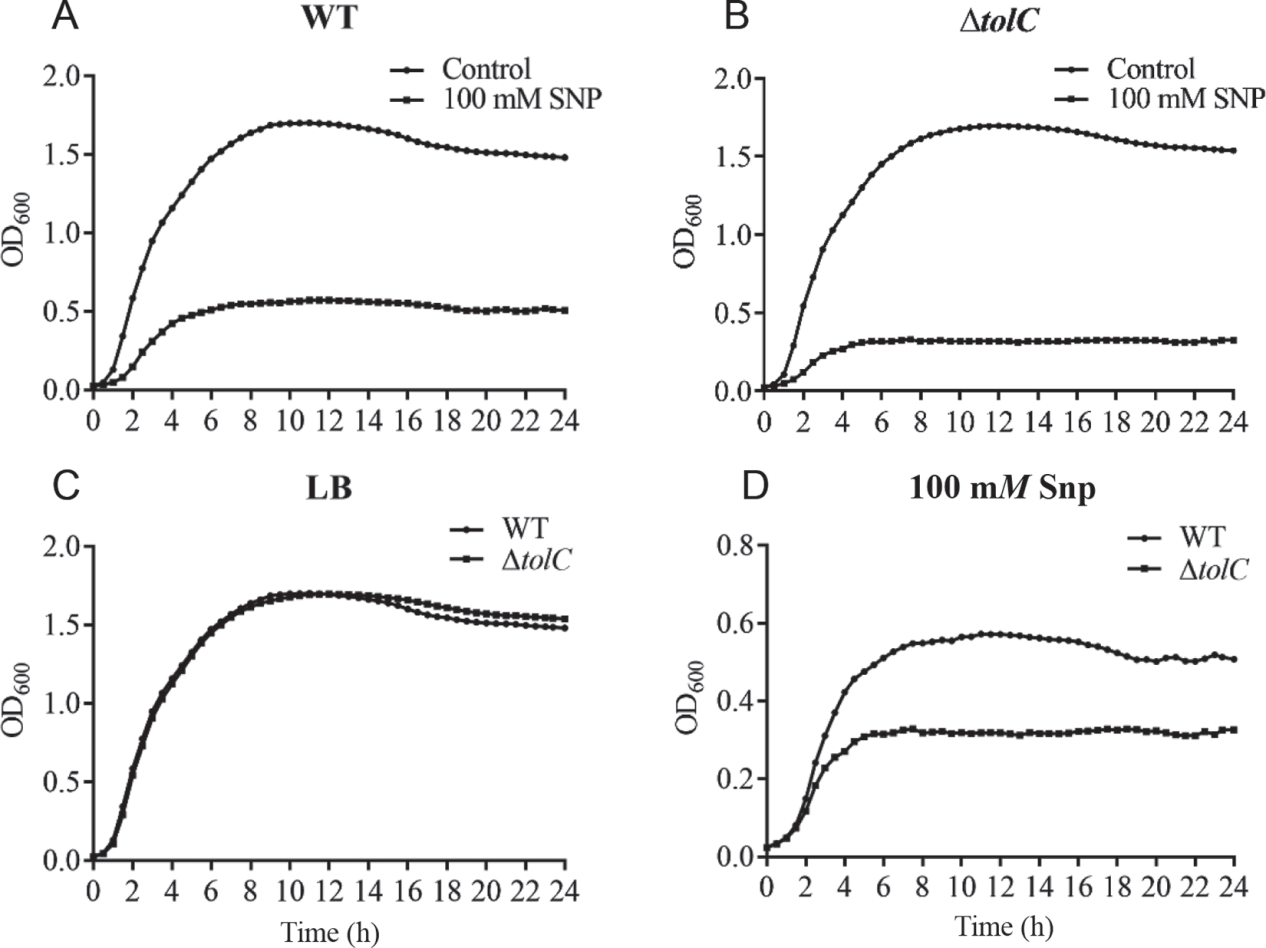
Growth curves (measured as optical density at 600 nm) of (A) *Cronobacter malonaticus* wild type (WT), and (B) *C. malonaticus* with deletion of the multiple efflux pump protein TolC (Δ*tolC* mutant) under 100 m*M* sodium nitroprusside (Snp) stress. Comparison of growth curves of 2 strains in (C) Luria-Bertani (LB) broth and (D) LB broth with 100 m*M* Snp. (E) Relative change in cell number was calculated as the number of cells in the LB broth divided by the number of cells in the cultures with 100 m*M* Snp for 0.5, 1.5, and 2.5 h, respectively. Error bars indicate standard deviations. Asterisks indicate difference between WT and mutant at *P < 0.05.

**Figure 2:**
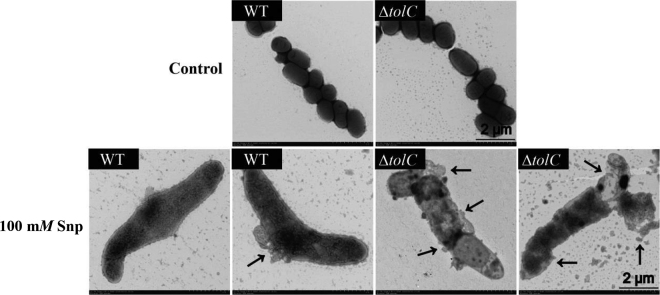
Morphological changes of *Cronobacter malonaticus* wild type (WT) and *C. malonaticus* with deletion of the multiple efflux pump protein TolC (Δ*tolC* mutant) under sodium nitroprusside (Snp) stress using transmission electron microscopy. The arrows indicate the sites of morphological injury to the bacteria.

Bacterial biofilms are a lifestage of some species in which cells are embedded in a self-produced matrix that is adherent to a surface (Flemming et al., 2016). In the food industry, biofilm formation is one of the critical factors in persistent contamination by foodborne pathogens (Ye et al., 2018). Biofilm forms a protective microbial barrier against various environmental stresses; thus, cells in biofilms are generally more resistant than planktonic cells to the same stressor or condition (Hall-Stoodley et al., 2004; Winkelströter et al., 2014). In this study, we assessed biofilm formation of strains by CVS assay, scanning electron microscopy, and CLSM. For CVS assay, biofilm formation by the WT strain was increased compared with that of the Δ*tolC* strain under normal conditions (without stressors), and inactivation of *tolC* impaired biofilm formation. Biofilm formation by both strains was reduced significantly under 100 m*M* Snp, although biofilm formation by WT was greater than that by Δ*tolC* when exposed to Snp (Figure 3A, B, C). Nitric oxide has been reported to enhance or decrease biofilm formation in different bacteria at different concentrations. In *E. coli*, 500 n*M* Snp could remove approximately 38% of biofilm, but the same concentration of Snp induced stronger dispersal of *Vibrio cholerae* and *Bacillus licheniformis* biofilm (Barraud et al., 2009). However, NO was shown to stimulate biofilm formation by controlling the levels of the bacterial secondary messenger cyclic diguanosine monophosphate (c-di-GMP) in *Shewanella oneidensis* (Plate and Marletta, 2012). In *P. aeruginosa* treated with low concentrations of Snp (25 n*M* to 2.5 m*M*), biofilm formation was decreased, whereas a high concentration of Snp (25 m*M*) enhanced biofilm formation (Barraud et al., 2006). In *Vibrio harveyi*, 50 n*M* NO promoted biofilm formation (Henares et al., 2013). Likewise, NO can disrupt *Staph. aureus* biofilms at high concentrations (125–1,000 μ*M*) but enhance biofilm formation at lower concentrations (0.975–1.96 μ*M*; Jardeleza et al., 2011). According to scanning electron microscopy (Figure 3E), biofilm did not form well after 24 h of incubation, and numerous planktonic cells were observed on the glass coverslip. The best ability to form biofilm by both strains occurred after culture for 48 h. At this stage, cells were gathered into a mass and the biofilms were compact and tight. After 72 h of incubation, biofilms of *C. malonaticus* dispersed. Biofilms were disrupted and reverted to the planktonic state when treated with Snp. In addition, we found that biofilms of Δ*tolC* were weaker and looser than those of the WT strain under the same Snp concentration. As shown in Figure 3F, mature biofilms formed after 48 h of incubation, as shown by green staining under CLSM. The numbers of dead cells (stained red) increased after 72 h in culture and were more prominent under treatment with 100 m*M* Snp than in the control group. As seen in the CVS assay and by scanning electron microscopy, biofilms of WT were stronger than those of Δ*tolC* under all circumstances. Bay et al. (2017) found that deletions of *tolC* resulted in significant reductions in biofilm-forming phenotype and enhanced antimicrobial susceptibilities in *E. coli.* In *Actinobacillus pleuropneumoniae*, loss of TolC impaired biofilm formation by reducing cell surface hydrophobicity and autoaggregation during the process of initial attachment (Li et al., 2016). Other types of efflux pump, such as MexAB-OprM of *P. aeruginosa*, AdeFGH of *Acinetobacter baumannii*, and AcrD of *S*. *enterica*, also play crucial roles in bioﬁlm formation (Alav et al., 2018). Together, our results and the current literature suggest that TolC is a critical component of the efflux pump and essential to biofilm formation. Nitric oxide can affect growth of bacteria and biofilm formation depending on the species and the concentration of NO. In this study, we investigated the characteristics of TolC under NO stress in *C. malonaticus* through phenotypic analysis; the molecular mechanisms of TolC in *C. malonaticus* remain to be revealed.

**Figure 3:**
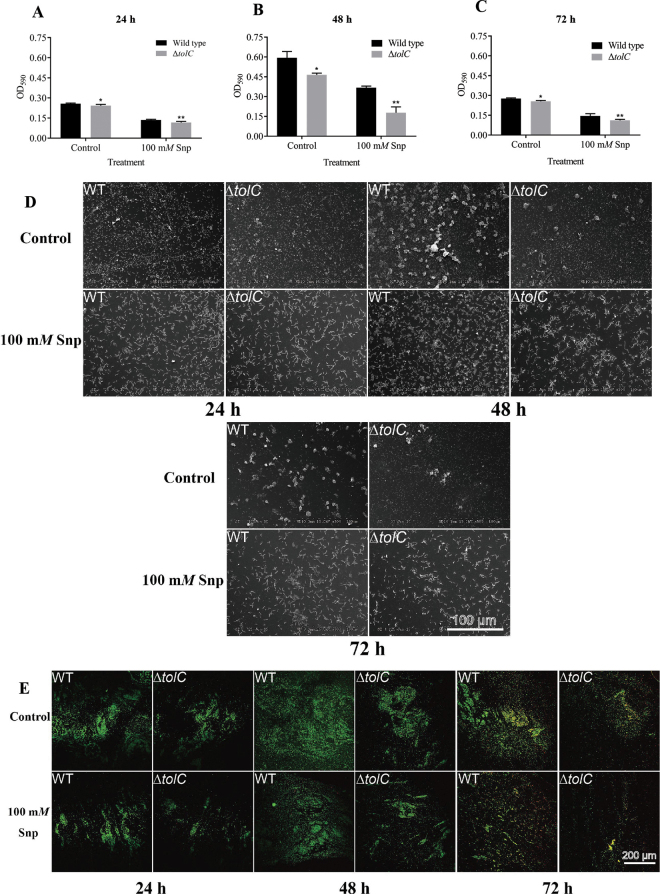
Biofilm formation by *Cronobacter malonaticus* wild type (WT) and *C. malonaticus* with deletion of the multiple efflux pump protein TolC (Δ*tolC* mutant) under 100 m*M* sodium nitroprusside (Snp) stress for (A) 24 h, (B) 48 h, and (C) 72 h using crystal violet staining assay, measured by optical density (OD) at 590 nm. Error bars indicate standard deviations. Asterisks indicate difference between WT and mutant at *P < 0.05 and **P < 0.01. Biofilm formation of *C. malonaticus* WT and *ΔtolC* under 100 m*M* Snp stress for 24, 48, and 72 h using (D) scanning electron microscopy and (E) confocal laser scanning microscopy.
